# Molecular Dynamics Simulation of Ligands from *Anredera cordifolia* (Binahong) to the Main Protease (*M*^pro^) of SARS-CoV-2

**DOI:** 10.1155/2022/1178228

**Published:** 2022-11-22

**Authors:** Jaka Fajar Fatriansyah, Ara Gamaliel Boanerges, Syarafina Ramadhanisa Kurnianto, Agrin Febrian Pradana, Fadilah, Siti Norasmah Surip

**Affiliations:** ^1^Department of Metallurgical and Materials Engineering, Faculty of Engineering, University of Indonesia, Depok, Jawa Barat 16424, Indonesia; ^2^Department of Medicinal Chemistry, Faculty of Medicine, Universitas Indonesia, Salemba Raya, Jakarta 10430, Indonesia; ^3^Faculty of Applied Sciences, Universiti Teknologi MARA, 40450 Shah Alam, Selangor, Malaysia

## Abstract

COVID-19 in Indonesia is considered to be entering the endemic phase, and the population is expected to live side by side with the SARS-CoV 2 viruses and their variants. In this study, procyanidin, oleic acid, methyl linoleic acid, and vitexin, four compounds from binahong leaves-tropical/subtropical plant, were examined for their interactions with the major protease (Mpro) of the SARS-CoV 2 virus. Molecular dynamics simulation shows that procyanidin and vitexin have the best docking scores of −9.132 and −8.433, respectively. Molecular dynamics simulation also shows that procyanidin and vitexin have the best Root Mean Square Displacement (RMSD) and Root Mean Square Fluctuation (RMSF) performance due to dominant hydrogen, hydrophobic, and water bridge interactions. However, further strain energy calculation obtained from ligand torsion analyses, procyanidin and vitexin do not conform as much as quercetin as ligand control even though these two ligands have good performance in terms of interaction with the target protein.

## 1. Introduction

Coronavirus disease (COVID-19) is an infectious disease caused by the SARS-CoV 2 virus. This disease was first reported to have spread in Wuhan, China, on December 31st, 2019. Less than three months later, on March 9th, 2020, COVID-19 was declared a pandemic by the WHO (World Health Organization). Until now, in August 2021, the total number of cases of this disease reached 601 million around the world, resulting in the deaths of approximately 6.49 million victims. Indonesia is one of the countries most affected by COVID-19. In Indonesia alone, 6.35 million cases have occurred since the pandemic started on February 2020, and it took 158 thousand lives in Indonesia [1]. Although in Jakarta, the Indonesian capital, COVID-19 was considered to be entering the endemic phase, where mortality is low [2], the countryside is still affected, and the population is expected to live side by side with the existence of the SARS-CoV 2 viruses and their variants [3].

There are two most common methods to explore COVID-19 antiviral medications, mainly through experiments and simulations. Experimental methods can be carried out in two ways: in vivo and in vitro. Meanwhile, the simulation method is called in silico. While experimental methods play a significant role in drug discovery, they can be time-consuming and costly [4]. Particularly when they are repeatedly employing the trial and error approach. To simulate the performance and efficacy of medications in treating COVID-19, the in silico method may be a preliminary step and can be very important to speed up drug discovery [5–9].

Tropical countries have abundant medicinal plant resources which have yet to be discovered [10–12]. The binahong (*Anredera cordifolia*) is a plant native to tropical and subtropical South American countries and later brought to Southeast Asia. It is abundant in Indonesia, especially on the island of Java. Binahong is considered an herb and has long been used as medicine. Binahongs abundance of antioxidants makes it potentially useful as a wound healer [13] or possibly as a cure for cataracts [14]. Binahong has a strong antioxidant activity and is frequently utilized by locals as a treatment because it includes flavonoid compounds in its leaves, stems, and flowers. In addition, this plant is frequently utilized to prevent the spread of related viruses like influenza [15].

In this study, procyanidin, oleic acid, methyl linoleic acid, and vitexin were four compounds from binahong leaves [14] examined for their interactions with the major protease (Mpro) of the SARS-CoV 2 virus. Mpro is a key enzyme crucial for the processing of polyproteins translated from viral RNA, and it has a vital role in the reproduction of SARS-CoV 2 and the release of many of its proteins [16, 17]. The investigation of how each compound/ligand inhibits the replication of SARS-CoV 2 viruses in this study is facilitated with molecular docking simulations, followed by molecular dynamics, toxicity tests, and drug feasibility studies. We prescreened the docking score using an online molecular docking service. It turned out that procyanidin, oleic acid, methyl linoleic acid, and vitexin were the most viable choices to be studied. This study's findings were compared with those of a control ligand, quercetin, which is effective at inhibiting Mpro SARS-CoV 2 [18]. In their publication, Agrawal et al. [19] stated that in vitro studies demonstrated that quercetin can interfere with multiple coronavirus entry and replication cycle steps.

## 2. Computational Methods

Molecular docking was carried out using the Maestro software's glide docking feature [20]. The Desmond function of the Maestro software was then used to run a molecular dynamics simulation for 20 ns with a trajectory record of 20 ps using an npt ensemble.

### 2.1. Ligand and Protein Preparation

Toxicology predictions for ligands and ADMET were also made using the pkSCM [21] and Molsoft websites with the hyperlink https://structure.bioc.cam.ac.uk/pkcsm and https://www.molsoft.com/, respectively. The three-dimensional structure of the ligands was obtained from the PubChem website https://pubchem.ncbi.nlm.nih.gov/. Meanwhile, the three-dimensional structure of SARS-CoV 2 Mpro was obtained from the Protein Data Bank (PDB) website https://www.rcsb.org/ with PDB ID 7L11 [22].

Initially, the LigPrep function of the Maestro application was used to prepare the three-dimensional structures of each ligand. During this preparation, hydrogen atoms are removed. Epik v2.9 was used to check for potential ionization states of ligands and tautomers in the pH range of 7 ± 2. Using the Protein Preparation Wizard function of the Maestro program, the three-dimensional structure of proteins is prepared by attaching hydrogen atoms, eliminating water molecules that do not interact with proteins, enhancing protein structure, and optimizing hydrogen bonds in proteins.

### 2.2. Molecular Docking

The grid area is determined using the receptor grid generation feature to identify the region in the system that serves as a receptor. The grid area is set using the receptor grid generation feature in the Maestro. The grid is set with the inhibitory center of XF-1, the native ligand of PDB ID 7L11, at the protein's active site. We locked a grid area with the center coordinates of *X* = 150.68, *Y* = 125.21, and *Z* = 233.34 with a box size of 20. In the Ligand section, the Van der Waals radius is scaled to 1, with a partial charge cutoff of 0.25. Molecular docking uses an extra-high level of accuracy (XP) option.

### 2.3. Molecular Dynamics Simulation

We used the feature XP Glide docking for molecular dynamics simulation, where the input is prepared and docked protein and ligands. The system builder feature is set by setting the solvent with the SPC (water molecule) model in the form of a cubic simulation box. The simulation box is calculated using the buffer method with a distance of *a* = 10 Å, *b* = 10 Å, and *c* = 10 Å. The next step is to minimize the solvent volume in which the ions section must be set to exclude the placement of ions and salts, which are 20 Å apart. Then 4Na^+^ ions and 0.15 M salt were added for neutralization. The system that has been set is then ready for molecular dynamics simulation with the Desmond Molecular Dynamics feature. Molecular dynamics simulation was carried out for 20 ns, with a trajectory record of 20 ps, in an npt ensemble [7]. In addition, we conducted ligand torsion analysis. Desmond feature provides internal torsional energy, which can be used to calculate strain energy using the following equation:

(1) $$\begin{array}{c} <E>=\frac{\sum_{\tau }\left[E\left(\tau \right)\exp\;\left(-E\left(\tau \right)/KT\right)\right]}{\sum_{\tau }\left[\exp\;\left(-E\left(\tau \right)/KT\right)\right]}\text{,}\\ Es=<E*>-<E>\text{.} \end{array}$$

where *E*(*τ*) is torsion energy, *k* is Boltzaman constant, *T* is temperature, 〈*E∗*〉 is energy for corresponding temperature, and <*E*> is the fitted energy. Detailed derivation and information can be obtained in [23].

### 2.4. ADMET Prediction

Initially, toxicological predictions of all ligands were carried out using the pkSCM and Molsoft websites to see if the ligands were safe if used as human drugs. Prediction is made by writing the SMILES string of the ligand compound and then selecting what properties are to be predicted for example absorption (water solubility, intestinal absorption, and skin permeability) distribution, metabolism, excretion, and toxicity. The molecular properties of each compound will then be used to determine the drug-likeness of the ligand compound through Lipinski's Rule of Five and the drug-likeness model score via the https://molsoft.com/mprop/page.

### 2.5. Residue Binding Site

**Figure 1:**
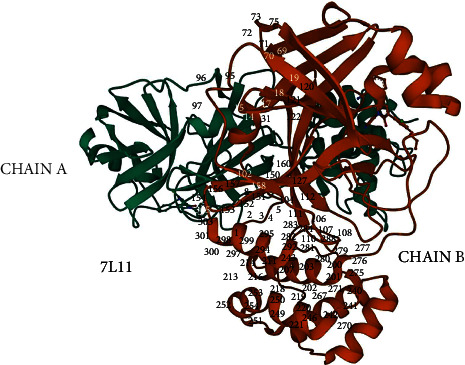
Residue binding site of protease enzyme of SARS-CoV 2 (PDB ID: 7L11).

**Figure 2:**
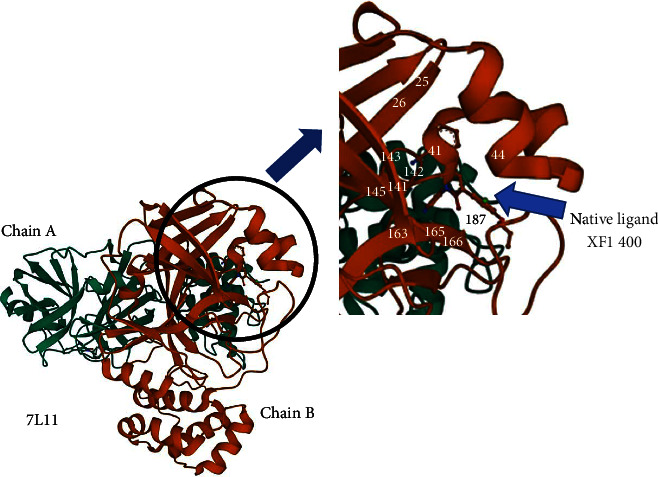
Residue binding site of main protease enzyme of SARS-CoV 2 (PDB ID: 7L11).

The binding site is a region on a protein macromolecule that binds to a specific ligand. The event of ligand binding to a protein can result in a change in the shape of the protein and can also change the function of the protein. In this study, the binding of the ligand to the viral protease enzyme can change the shape of the protein and render the protein unable to help viral replication in human cells. In this study, it is necessary to identify any sites on the protein that can become binding sites. There are two ways to determine the binding site, namely, by looking directly at the official website of the Protein Data Bank (via the rcsb.org website), and the second way is to use the SiteMap feature in the Maestro Schrödinger application. The data obtained are in the form of protein residues that become binding sites, as shown in Tables 1 and 2 and the map is shown in Figures 1 and 2.

## 3. Results and Discussion

### 3.1. Lipinski's Rule of Five, Druglikeness Score, and ADMET Prediction

The results of Lipinski's rule and Druglikeness scores are shown in Table 3 and ADMET predictions are shown in Table 4.

Except for procyanidin, every ligand in Table 3 complies with Lipinski's rule because this ligand has a high violation score. Procyanidin has been proven to show beneficial pharmacological properties, including antioxidant, antibacterial, and anti-inflammatory effects [24]. This molecule has a molecular weight greater than 500 and hydrogen interactions greater than 5. Other ligands, however, are typically safe to use as drugs. Lipinski's rule is not the only factor in assessing whether a molecule is suitable for use as a drug. Even though many compounds fail to follow the Lipinski rules and are predicted to have low bioavailability, they are frequently utilized as medications on the market [25]. The limits of Lipinski's rules demand a more thorough drug feasibility investigation, specifically the prediction of ADMET properties.

Table 4 shows the results of the ADMET prediction. The information in the table is then analyzed using the theory detailed on the pkSCM website https://structure.bioc.cam.ac.uk/pkcsm. All ligands are fairly water-soluble, which qualifies them for drug usage and allows for simple dissolution in the body. The very modest maximal dosage further supports this for these two ligands. Oleic acid and methyl linoleic acid ligands have the lowest volume of distribution (VDss) (measured in log L/kg) of all the ligands when it comes to distribution. Oleic acid and methyl linoleic acid ligands are CYP2D6 substrates and inhibitors based on their metabolic characteristics, which can affect how the body processes drugs because CYP2D6 and CYP3A4 cytochromes are crucial for this process. In excretion properties, the total clearance value of each ligand was in the medium category (0.3–0.7) except for methyl linoleic acid and oleic acid ligands with low total clearance values, which suggests that the kidneys have trouble excreting methyl linoleic acid and oleic acid ligands. The last analysis is related to toxicity; all ligands passed the ADMET toxicology test. However, methyl linoleic acid and oleic acid ligands have skin sensitization properties that can harm the skin and several layers of tissue when these drugs are in the body.

The ADMET prediction offers more precise forecasts for each ligand's toxicity and pharmacology. The reasoning, as mentioned earlier, leads to the conclusion that, compared to other ligands, methyl linoleic acid and oleic acid ligands tend to be inappropriate for drug use. However, we acknowledge that the drug-likeness score is only partial. Even with negative prediction, we still consider those compound to be docked.

### 3.2. Molecular Docking Results

Molecular docking simulations were performed for each test ligand (an herbal compound from the binahong plant) and quercetin as the control ligand. The data obtained are the molecular docking score (docking score) and binding energy [26]. The data obtained as a result of the molecular docking simulation is shown in Table 5.

The ligands are sorted by molecular docking score. A relatively small molecular binding score indicates a stable bond between the ligand and the Mpro virus target protein. A lower molecular binding score indicates an optimal binding site and binding energy. In addition to the molecular docking score (or docking score), the binding energy (bind energy) score is also obtained using the MMGBSA feature in the Maestro program. MMGBSA is one of the most popular methods for calculating the free energy involved in ligand binding to target proteins [27, 28]. The energy scores obtained by the MMGBSA indicate that the smaller the energy score, the stronger the ligand and protein binding [29]. Based on the docking score, procyanidin has the best molecular docking scores, followed by vitexin, quercetin, methyl linoleic acid, and oleic acid. However, based on the binding energy scores, methyl Linoleic acid has the lowest binding energy score, followed by procyanidin, oleic acid, vitexin, and quercetin. Thus, procyanidin demonstrates better bonding performance with target proteins than other ligands. These results are not conclusive yet since both docking and MMGBASA scores can be used independently as scoring functions to predict binding affinity and ligand performance. The extent of our calculation deviation for the docking score is 0.001.

**Figure 3:**
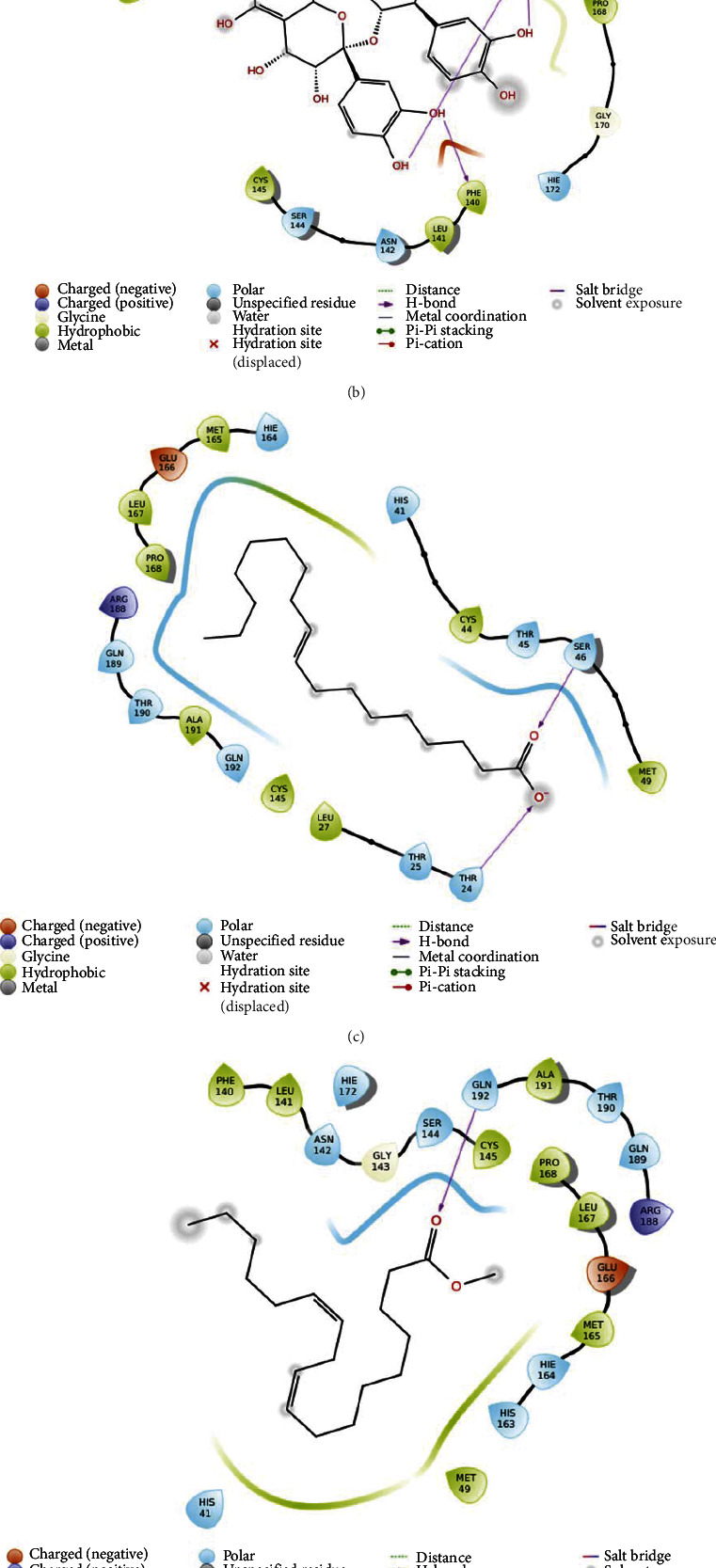
Ligand-protein 2D interaction diagram of molecular docking. (a) Quercetin (control), (b) procyanidin, (c) oleic acid, (d) methyl linoleic acid, and (e) vitexin.

The 2D simulation of each ligand with the protein target is shown in Figure 3.

### 3.3. Molecular Dynamics Results

The stability of docked complexes and the binding pose obtained in docking studies are widely verified by molecular dynamics simulation studies. The molecular dynamic simulation yields Root Mean Square Deviation (RMSD), Root Mean Square Fluctuation (RMSF), and Protein-Ligand Contact.

### 3.4. Root Mean Square Deviation (RMSD)

**Figure 4:**
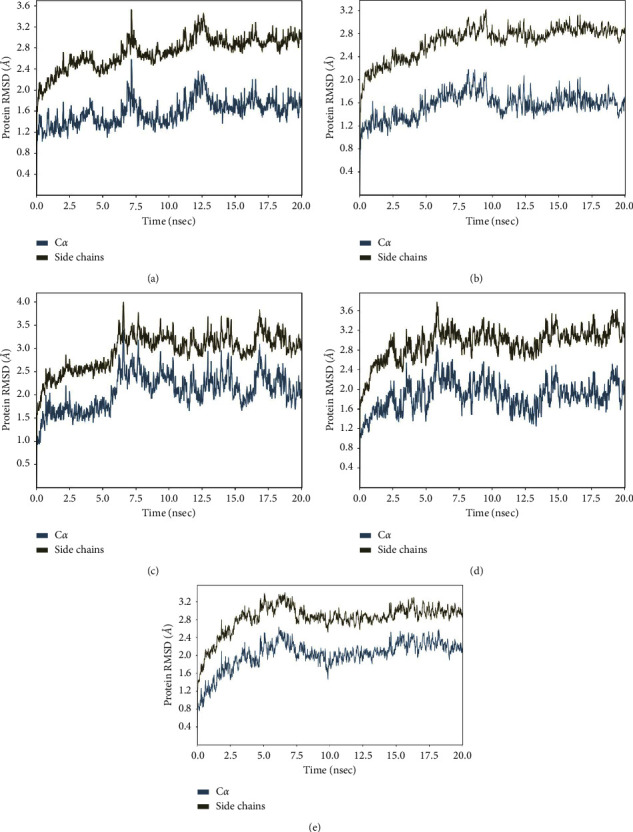
RMSD plots for each ligand: (a) quercetin; (b) procyanidin; (c) oleic acid; (d) methyl linoleic acid; (e) vitexin.

RMSD is an atom's average movement or displacement at a specific time interval. In other words, RMSD is data that shows the average distance between atoms (ligands) and proteins. The RMSD plot for each ligand is shown in Figure 4.

The +20 ps (0.02 ns) timestep, or the start of the simulation, saw a significant increase in the concentration of each ligand. The red plot for each ligand displays the RMSD value for the alpha carbon (C-*α*), which is observed. The interaction between ligand and protease is considered stable when the RMSD value of the backbone is under 2.5 Å [30]. The average C-*α* RMSD value for the quercetin control ligand is below 2.5 Å and is stable throughout the simulation, as shown in Figure 4(a). Overall, the quercetin control ligand's average RMSD value was 2.15 Å. Procyanidin and vitexin ligands both have an average RMSD value for alpha carbon <2.5 Å, for procyanidin, it is 2.08 Å, while for vitexin, it is 2.05. Meanwhile, the RMSD values for the ligands methyl linoleic acid and oleic acid tend to fluctuate throughout the simulation, with an average RMSD value of 2.5 Å for methyl linoleic acid and 2.51 Å for oleic acid.

According to the decreasing value of RMSD, the procyanidin ligand has the lowest average RMSD value. Then, followed by quercetin (control ligand), vitexin, methyl linoleic acid, and oleic acid. Thus, procyanidin, quercetin, and vitexin have a better binding interaction with the target protein compared to the other ligands since the lower RMSD value denotes that the binding of the ligand to the target protein is relatively stable. Procyanidin is an ACE (angiotensin I converting enzyme) activity inhibitor due to tetramer, and its inhibition activity depends on its structure [31]. Although ACE and ACE-2 (which are important in SARC-COV 2) are not related directly, inhibiting ACE reduces the production of Angiotensin-2, which is important to the ACE-2 mechanism [32].

**Figure 5:**
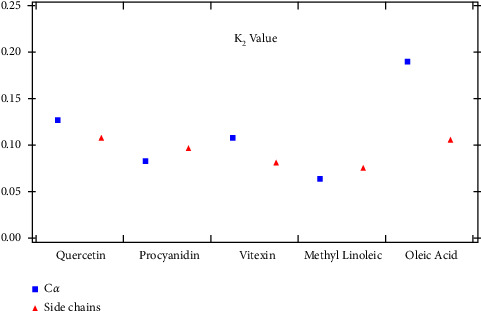
*K*_2_ coefficient value of fitted RMSD.

To make sure that ligands are stable, we conducted fitting, which assumes that RMSD behaves like a power function. The fitting equation is given by the following equation:

(2) $$\begin{array}{c} RMSD\left(t\right)=K_{1}t^{K_{2}}\text{,} \end{array}$$

where *K*_1_ and *K*_2_ are arbitrary constants. The lower value of *K*_2_ demonstrates more stable ligands. The *K*_2_ coefficient value of fitted RMSD is given in Figure 5. The plot shows that Procyanidin, Vitexin, and Methyl linoleic acid have a relatively lower value of *K*_2_ coefficient which demonstrates higher stability than other ligands.

### 3.5. Root Mean Square Fluctuation (RMSF)

**Figure 6:**
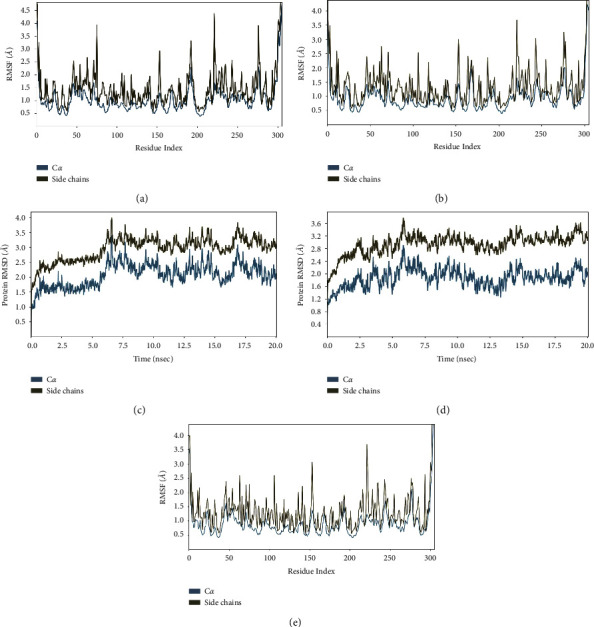
RMSF plots for each ligand: (a) quercetin; (b) procyanidin; (c) oleic acid; (d) methyl linoleic acid; (e) vitexin.

RMSF measures how far atomic locations have deviated from the initial point. In other words, RMSF illustrates how dynamic the protein-ligand interaction is. Similar characteristics can be seen in the RMSF plot of ligand-protein for each ligand, where the RMSF value at the *N*-terminal and *C*-terminal residues (start and end) is very high because these residues represent the tails or ends of the protein structure, which are free to move and highly reactive. The RMSF plots are displayed in Figure 6 for each ligand.

The RMSF plot produces a plot with peaks. The fluctuation value is high throughout the simulation since each peak denotes a high RMSF value. In this analysis, ligand and protein interactions with RMSF values above 2.5 Å are considered to have bonds that are less stable.

Figure 6(a) shows the RMSF analysis for the quercetin control ligand. The total number of residues with a high RMSF value (RMSF value >2.5 Å) was 9, three of which were protein binding sites. There are ten residues for procyanidin ligands with high RMSF, and five of these are protein binding sites. The oleic and methyl linoleic acid ligands have seven residues with high RMSF values, of which three are protein binding sites. Four of the seven residues in the vitexin ligand, which has a relatively high RMSF value, are binding sites. According to the data above-given, the procyanidin ligand contains ten peaks, ten of which have an RMSF value of >2.5 Å. Quercetin had nine peaks, followed by vitexin, methyl linoleic acid, and oleic acid, each of which had seven peaks. Five out of ten residues are binding sites for the procyanidin ligands. Four of seven binding site residues for vitexin ligands had RMSF values of more than 2.5 Å. Oleic acid had 4 out of 7 residues, while methyl Linoleic acid had just 3 out of 7 residues. The control ligand quercetin had only 3 out of 9 residues. The target protein binds to the control ligands quercetin > methyl linoleic acid > vitexin and oleic acid > procyanidin, according to the results of the RMSF analysis.

### 3.6. Protein-Ligand Contact

The interaction simulation diagram is a bar chart representing the bond of each residue with the ligand and the type of bond formed between the residue and the ligand. The plot's *x*-axis is the residues sorted by an index number, and the *y*-axis is the interaction fraction of each bond between the protein and ligands. The bonds in the target ligand-protein are hydrogen interactions, hydrophobic contacts, ionic interactions, and water bridges.

**Figure 7:**
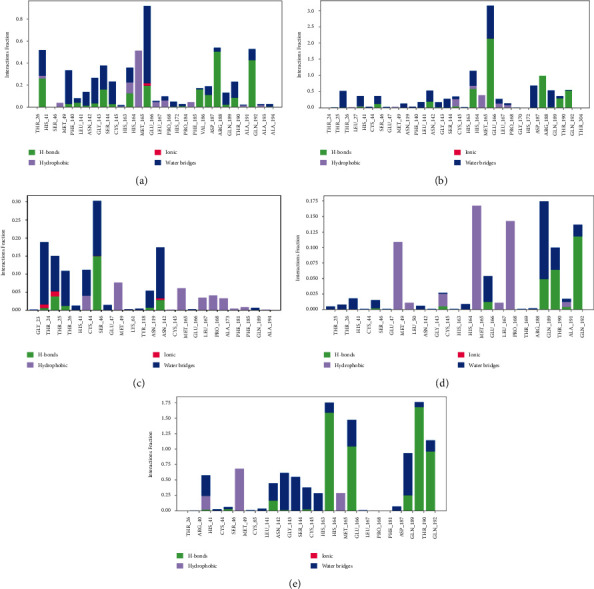
The simulation interaction of each ligand with the protein target: (a) quercetin (control), (b) procyanidin, (c) oleic acid, (d) methyl linoleic acid, and (e) vitexin.

The hydrogen interaction is the most important bond in the simulation because this type of bond is very strong and plays a very important role in the determination and specification of ligands as drugs. Another dominant interaction is the hydrophobic interaction that occurs when both the ligand and the residues repel water (hydrophobic) so that they bind to each other. Hydrophobic interaction is indicated by a green bar plot, while a purple bar plot indicates hydrophobic interactions. Ionic interactions occur in polar ligands and polar residues. A red bar plot indicates these interactions. Water bridge interactions occur at residues and ligands that interact with hydrogen and are mediated or mediated by water. A blue bar plot indicates this interaction. The interaction between each test ligand and the control quercetin ligand is shown in Figure 7.

The *x*-axis on the plot shows the residues interacting with the ligand, and the *y*-axis on the plot shows the bond fraction. For example, the 0.3 fraction shows that +30% of these interactions occur during the simulation time. However, in the plot of some ligands, there are several residues whose fraction value is greater than 1, which indicates that these residues have several contacts at once.

Overall, procyanidin ligands had the most interactions with residues among other ligands, with a reasonably high interaction fraction, reaching number 3, and it was followed by control ligands quercetin, vitexin, oleic acid, and methyl linoleic acid. Procyanidin ligands, control quercetin, and vitexin have a dominant hydrogen interaction, making the interaction between these ligands and the target protein very strong. In addition, interactions with water molecules (water bridges) are also quite dominant.

Meanwhile, oleic acid and methyl linoleic acid ligands have low interaction fractions. The interaction of these ligands is also not as significant as the other three, which in turn, makes the interaction of these two ligands with the target protein poor. Overall, the most dominant interactions are hydrogen, hydrophobic, and water bridge interactions.

### 3.7. Ligand Torsion Analysis

Each ligand has a single, rotatable bond. In molecular dynamics simulation, ligands as chemical compounds move without breaking the chemical bonds. One of the movements that occur is rotamer, where this movement is in the form of rotation that occurs in single bonds in the ligand [33]. This ligand torsion conformation can provide information on how well the ligand torsion is based on theoretical calculations and the position the ligand tends to desire throughout the simulation [23]. The value of internal torsional energy is needed to see the strain energy present in the ligand. Strain energy is the total energy of the molecule. This strain energy can explain how easily or not the ligand conforms. The strain energy value is obtained by equation (2).

From the calculation results of each rotatable bond on each ligand, the internal torsional energy values are shown in Table 6.

Table 6 indicates the smallest strain energy values indicated by the methyl Linoleic acid ligand and the control ligand quercetin, followed by oleic acid, vitexin, and procyanidin, which had the highest strain energy value. When the ligand binds to a protein, the conformation of the ligand will experience various disturbances (rotational movement) that come from the ligand's binding to the protein itself. Protein and ligand interactions can be either attractive or repulsive. Due to this ligand-protein interaction, the conformation of the ligand will be seen to fluctuate at higher temperatures [23], and this phenomenon is explained by parameter *b* in equation 4.4 [34].

Strain energy indicates whether a ligand has Procyanidin and vitexin ligands have a higher strain energy value than the control ligand quercetin, which indicates that these two ligands do not conform as much as the quercetin and the interaction between the ligand and target protein is relatively stable. Based on the results of molecular docking and molecular dynamics analysis, both procyanidin and vitexin have good performance in terms of interaction with the target protein. This proves that procyanidin and vitexin ligands do not form as much conformation as quercetin control ligands when interacting with target proteins, as seen from the *E*_*s*_ values of these ligands, which are more favorable (positive) than control quercetin ligands and it was found that many conformations to form stable interactions with the target protein [23].

## 4. Conclusions

Overall, all ligands complied with Lipinski Rule of Five, except for procyanidins. From the score, methyl linoleic acid and oleic acid are not eligible because they have negative scores. Thus, two compounds are not eligible to be used as a drug. ADMET predictions also support that these ligands are not suitable for drug use. Based on molecular docking scores and binding energy, procyanidin, vitexin, and quercetin control ligands demonstrated stronger interactions than methyl linoleic acid and oleic acid ligands. Molecular dynamics simulation showed that procyanidin had the best RMSD and RMSF performance of all ligands, followed by vitexin and quercetin control ligands. From this simulation, it is known that the dominant interactions in each ligand and target protein are hydrogen, hydrophobic, and water bridges interactions. In addition, the strain energy calculation shows that even though procyanidin and vitexin have good performance in terms of interaction with the target protein, they have a higher strain energy value than the control ligand quercetin, which indicates that these two ligands do not conform as much as the quercetin. However, overall ligands extracted from binahong leaves have good performance in terms of molecular dynamics and docking score results comparable to quercetin inhibiting Mpro SARS-CoV 2.

## Figures and Tables

**Table 1:** Residue binding site of protease enzyme of SARS-CoV 2 (PDB ID: 7L11) which is calculated using Maestro Schrodinger.

Residues binding site							
**1**	2	3	4	5	8	14	15
**17**	18	19	31	69	70	71	72
**73**	75	95	96	97	102	104	106
**107**	108	109	110	111	112	119	120
**121**	122	127	132	150	151	152	153
**154**	156	157	158	160	200	201	202
**203**	207	211	213	214	216	218	219
**220**	221	240	241	242	246	249	250
**251**	252	253	254	267	270	271	274
**275**	276	277	279	280	281	282	283
**284**	286	288	291	292	293	294	295
**297**	298	299	300	301	303	305	634

**Table 2:** Residue binding site of main protease enzyme of SARS-CoV 2 (PDB ID: 7L11) which is derived from the PDB site [22].

Native ligand	Residue binding site						
XF1	25	26	41	49	140	141	142
	143	144	145	163	165	166	187

**Table 3:** Lipinski's rule of five and druglikeness score.

Ligand	Lipinski's rule of five		Druglikeness score
	Violation	Druglikeness	
Procyanidin	3	No	0.91
Methyl linoleic acid	1	No	−1.04
Oleic acid	1	No	−0.30
Vitexin	1	No	0.6
Quercetin	0	No	0.52

**Table 4:** Prediction results of ADMET properties of each ligand.

Properties		Procyanidin	Methyl linoleic acid	Oleic acid	Vitexin	Quercetin
Absorption	Water solubility (log ml/L)	−2.892	−6.3	−5.924	−2.845	−2.925
	Intestinal absorption (%)	55.27	91.222	91.823	46.695	77.207
	Skin permeability (log *K_p_*)	−2.735	−2.725	−2.725	−2.735	−2.735
Distribution	VDss (log L/kg)	0.193	−0.487	−0.558	1.071	1.559
	Fraction unbound	0.284	0.062	0.052	0.242	0.206

Metabolism	CYP2D6 substrate	No	No	No	No	No
	CYP3A4 substrate	No	Yes	Yes	No	No
	CYP2D6 inhibitor	No	No	No	No	No
	CYP3A4 inhibitor	No	No	No	No	No
Excretion	Total clearance	0.58	1.918	1.884	0.444	0.407
	Renal OCT2 substrate	No	No	No	No	No

Toxicity	AMES toxicity	No	No	No	No	No
	Max. tolerated dose	0.438	−0.689	−0.81	0.577	0.499
	Oral rat acute toxicity (LD50)	2.482	1.476	3.259	4.635	2.471
	Hepatotoxicity	No	Yes	No	No	No
	Skin sensitization	No	Yes	Yes	No	No

**Table 5:** Docking scores and MM-GBSA binding free energies.

Ligand	Docking score (kcal/mol)	Bind energy (kcal/mol)
Procyanidin	−9.132	−66.7
Vitexin	−8.433	−53.4
Quercetin (control ligand)	−7.474	−51.69
Methyl linoleic acid	−3.58	−77.65
Oleic acid	−3.092	−63.57

**Table 6:** Results of the calculation of the energy strain of each ligand.

Ligands	*E* (*τ*) (kcal/mol)	$\bar{b}$	*E*_*s*_ ligan (kcal/mol)
Procyanidin	1.094	1.67	3.82
Methyl linoleic acid	0.84	2.99	−0.39
Oleic acid	0.42	1.85	−0.00641
Vitexin	1.25	2.22	0.082
Quercetin (control ligand)	1.44	2.50	−0.22

## Data Availability

The data that support the findings of this study are available from the corresponding author upon reasonable request.
